# Enhanced Photocatalytic Performance under Ultraviolet and Visible Light Illumination of ZnO Thin Films Prepared by Modified Sol-Gel Method

**DOI:** 10.3390/molecules29174005

**Published:** 2024-08-24

**Authors:** Radka Gegova-Dzhurkova, Diana Nesheva, Irina Stambolova, Katerina Zaharieva, Valeri Dzhurkov, Ilko Miloushev

**Affiliations:** 1 G. Nadjakov Institute of Solid State Physics, Bulgarian Academy of Sciences, 1784 Sofia, Bulgaria; radka.dzhurkova@gmail.com (R.G.-D.);; 2 Institute of General and Inorganic Chemistry, Bulgarian Academy of Sciences, 1113 Sofia, Bulgaria; 3 Institute of Mineralogy and Crystallography “Acad. Ivan Kostov”, Bulgarian Academy of Sciences, 1113 Sofia, Bulgaria

**Keywords:** zinc oxide, thin films, sol-gel, hot air drying, malachite green, photocatalysis, photo-corrosion, lattice defects

## Abstract

Semiconductor oxides are frequently used as active photocatalysts for the degradation of organic agents in water polluted by domestic industry. In this study, sol-gel ZnO thin films with a grain size in the range of 7.5–15.7 nm were prepared by applying a novel two-step drying procedure involving hot air treatment at 90–95 °C followed by conventional furnace drying at 140 °C. For comparison, layers were made by standard furnace drying. The effect of hot air treatment on the film surface morphology, transparency, and photocatalytic behavior during the degradation of Malachite Green azo dye in water under ultraviolet or visible light illumination is explored. The films treated with hot air demonstrate significantly better photocatalytic activity under ultraviolet irradiation than the furnace-dried films, which is comparable with the activity of unmodified ZnO nanocrystal powders. The achieved percentage of degradation is 78–82% under ultraviolet illumination and 85–90% under visible light illumination. Multiple usages of the hot air-treated films (up to six photocatalytic cycles) are demonstrated, indicating improved photo-corrosion resistance. The observed high photocatalytic activity and good photo-corrosion stability are related to the hot air treatment, which causes a reduction of oxygen vacancies and other defects and the formation of interstitial oxygen and/or zinc vacancies in the films.

## 1. Introduction

Many industries discharge a large amount of water pollutants such as dyes, heavy metals, pesticides, etc. Most of the organic dyes are known to be toxic and/or carcinogenic and cause problems for humans and other live organisms. In particular, Malachite Green (MG), which is used as a dye for fabric, leather, and paper, is highly toxic and has to be purified before releasing the wastewater into the environment. MG poisons the human liver, causes damage to the kidneys, and may lead to cancer when inhaled, etc. [1,2]. A great number of techniques for decontamination of this wastewater have been suggested; among them, heterogeneous photocatalysis has attracted much interest due to its ability to effectively decompose many pollutants [3]. 

Photocatalysis is a phenomenon occurring when illuminating a semiconductor with ultraviolet (UV) or visible (VIS) light—electrons are excited from the valence band or defect states in the band gap and transferred to the conduction band. Thus, electron–hole pairs are created, which can either recombine or take part in the generation of free radicals that are further involved in oxidation-reduction reactions. For high photocatalytic activity, the density of the photogenerated carriers has to be high, and the rate of recombination of generated electron–hole pairs has to be low. The reuse of the photocatalysts is also very important for their practical application. 

It is important to use eco-friendly photocatalytic materials, which will have less environmental and ecological impact. Therefore, in recent years, semiconducting oxides (TiO_2_, ZnO, WO_3_, CuO, etc.) have received increasing attention as materials with great potential in the photocatalytic removal of various pollutants in wastewater. The most commonly used metal oxide photocatalysts are anatase TiO_2_ and wurtzite ZnO [4]. They are environmentally sustainable, safe, and low-cost. Nanosized ZnO particles are considered very promising photocatalysts since they can be prepared with various morphologies and sizes and demonstrate high photocatalytic efficiency due to their large total surface and high surface reactivity related to many active sites [3]. Unfortunately, in water solutions, ZnO reacts with water molecules, which causes considerable photo-corrosion (photo-dissolution) plus water contamination with Zn^2+^ ions [5,6]. This decreases the photocatalytic efficiency of ZnO and limits its reusability. Doping with rare-earth metals, deposition of another metal-oxide layer on the ZnO surface, ZnO/carbon nanoparticle hybridization, control of the solution’s pH, and engineering of intrinsic defects, etc., have been suggested for the inhibition of this important problem [5,7,8,9]. However, the functional groups improving the stability of the catalyst may also contaminate purified water. Furthermore, the photocatalytic activity of undoped nanosized ZnO is mainly in the UV region, and the recombination of photogenerated electrons and holes (e^−^/h^+^) is relatively rapid, which decreases the photocatalytic efficiency and still hinders the widespread use of nanosized ZnO in photocatalysis. Various approaches have been applied to increase the UV photocatalytic activity of undoped ZnO, as well as to extend its light absorption into the visible range without increasing the recombination of photogenerated carriers [5,7,10]. Doping with rare earth ions, noble and transition metals, surface and defect engineering, preparation of heterojunctions, and nanocomposites are among them [3,4,11,12,13,14,15,16,17,18]. 

The photocatalytic performance of ZnO thin films is restricted by their small surface area, but they are still interesting since the films can be coated on various substrates and their use does not require post-treatment to remove the catalyst. For the practical use of the layers, their photocatalytic activity must be high, and this motivates research by a significant number of researchers. The modification of widely applied preparation methods (sol-gel, hydrothermal, co-precipitation, wet chemical, etc.) and the application of green preparation techniques are among the tools for achieving better photocatalytic performance.

The investigations of the application of nanocrystalline ZnO films to the degradation of Malachite Green azo dye are rather limited in comparison with those performed on Methylene Blue and Methyl Orange [11]. In this study, the photocatalytic activity of two groups of undoped sol-gel nanocrystalline ZnO thin films in MG degradation is compared and discussed. The films were prepared by applying two different drying procedures—conventional furnace drying in static air at 140 °C or the two-step drying procedure that we have proposed recently [19]. The two-step drying procedure starts with treatment with hot air flow followed by furnace drying at 140 °C. Films from both groups were annealed post-deposition at 400 °C. It has been found that the hot air treatment affects the surface morphology and crystallinity of the films, significantly improves their photocatalytic activity during MG degradation in water solution under UV or VIS light illumination and makes possible multiple photocatalytic uses of the hot-air-treated films under both lights. These observations are explained assuming that the hot air treatment facilitates changes in the density of lattice defects, such as oxygen vacancy, interstitial oxygen, and zinc vacancy.

## 2. Results and Discussion 

### 2.1. Surface Morphology, Lattice Structure and Optical Properties

**Figure 1:**
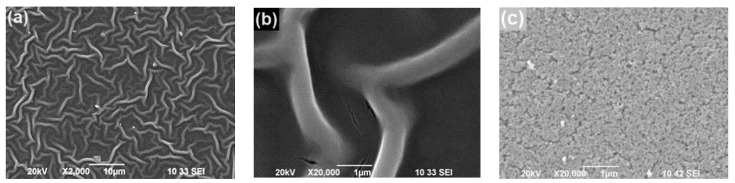
SEM surface images of I-2 film prepared by one-step furnace drying taken at: (**a**) magnification 2000×, (**b**) magnification 20,000×; (**c**) SEM surface image of II-2 film taken at magnification 20,000×. Both films were post-deposition annealed for 60 min in air at 400 °C.

The photocatalytic processes occur on the film surface, and the surface morphology strongly affects the photocatalytic performance. Information about the surface morphology of the ZnO films investigated here was obtained using scanning electron microscopy (SEM). Studies on samples from the two groups were carried out: Group I—sample I-1 was produced by using furnace drying in static air at 140 °C for 15 min, and sample I-2 was post-deposition annealed in air at 400 °C for 60 min; Group II—sample II-1 was prepared using the above-mentioned two-step drying procedure (treatment with hot air flow with temperature *T*_h_ = 90–95 °C for 5 min followed by drying in static air at 140 °C for 15 min) and sample II-2 was post-deposition annealed in air at 400 °C for 60 min. Details about the film preparation are given in Section 3.1, Figure 1a,b presents the SEM surface images of sample I-2 at two different magnifications; Figure 1c depicts an SEM surface image of sample II-2. Fiber-like structures (called wrinkles, stripes, ganglia) are seen on the surface of sample I-2; very similar structures have also been observed on the surface of the I-1 film. On the other hand, the surface of the annealed sample II-2 from Group II is uniform; the surface of the as-prepared film from this group (sample II-1) is also uniform. Pores of small size and relatively low density are observed on the surface of Group II films; one can think that the film bulk is also of relatively low porosity. 

During the drying of sol-gel ZnO films deposited on substrates with different chemical natures, shrinkage may take place due to the evaporation of organic remains, resulting in the appearance of internal stress in the films. When the evaporation rate of organic remains is high, the stress is high and may cause the appearance of wrinkles on the ZnO film surface [20,21,22]. The presence of monoethanolamine (MEA) in the sol plays an essential role in surface wrinkling due to its low vapor pressure, which affects the sol fluidity. We used ethanol and MEA for the sol preparation. The boiling temperature of ethanol is low, and the MEA evaporation starts at ~120 °C. It can be assumed that during the furnace drying of Group I films at 140 °C, the fast evaporation of the solvent and MEA occurs, high internal stress appears, and, therefore, wrinkles are formed on the films’ surface (Figure 1a,b). It is possible that during the treatment of Group II films with hot air flow (*T_h_* ≈ 90–95 °C) directed perpendicularly to the film surface, the solvent and MEA evaporation is slower than in the case of furnace drying. Thus, the film shrinkage will be retarded and the level of internal stress will be low, which will result in a uniform film surface. Therefore, lower lattice strain and defect density can be expected in the Group II ZnO films. Indeed, a careful analysis of the XRD results reported in [19] has revealed that in both as-prepared (II-1) and annealed (II-2) films produced by applying hot air treatment, the strain along the c-axis is approximately zero, while the films prepared by using convenient furnace drying exhibit significant tensile strain along the c-axis (0.24% [I-1] and 0.16% [I-2]). This can explain the different surface morphology seen in Figure 1.

**Figure 2:**
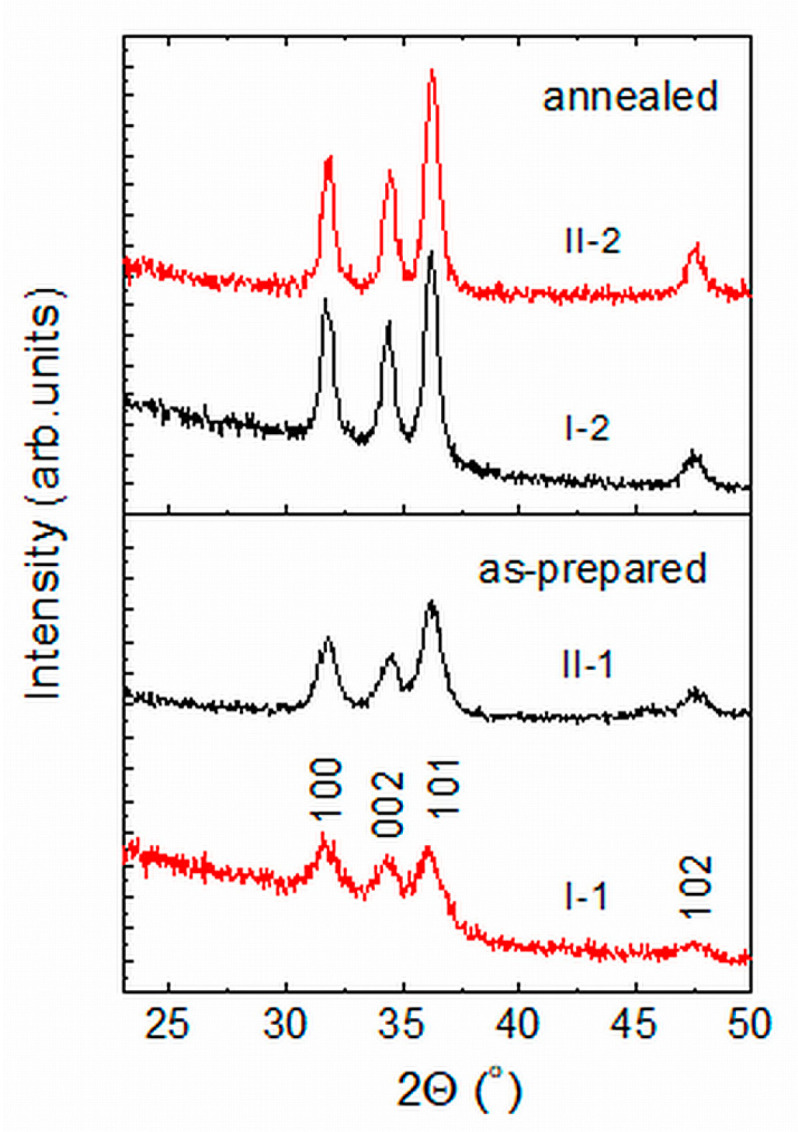
XRD patterns of as-prepared and annealed ZnO samples from Group I (I-1, II-1) and Group II (II-1, II-2). The patterns have been vertically shifted for clarity.

Information about the crystal structure and lattice order of the films was obtained through X-ray diffraction (XRD) studies. Figure 2 shows the XRD patterns of as-prepared and annealed ZnO films from Group I and Group II. Four characteristic peaks typical for crystalline ZnO with wurtzite structure (JCPDS No.36-1451) are seen in the spectra of all samples. A broad band centered at 2Θ ∼ 23° is also observed, which is due to diffraction from the amorphous SiO_2_ film grown on the silicon substrate. Looking at the spectra of the as-prepared films, one can notice that the XRD peaks related to ZnO are stronger in the spectrum of sample II-1 than in the spectrum of sample I-1. This implies that the usage of hot air treatment improved the crystallinity and reduced the density of lattice defects in the as-prepared films from Group II. By decomposing the segment of the XRD patterns in the 2Θ = 29°–39° range into three Lorentzians and applying Sherrer’s equation, values of *d* ≈ 7.5 nm and *d* ≈ 10 nm have been determined for the nanocrystallite diameter, *d*, in as-prepared Group I and Group II films, respectively [19]. For the annealed films, the *d*-values obtained were *d* ≈ 14.2 nm and *d* ≈ 15.7 nm for samples (I-2) and (II-2). Hence, the total area of the inter-grain interfaces in the II-1 films is smaller than in the I-1 films and, therefore, the density of crystal defects in the II-1 films is lower than in I-1. In addition, the hot air treatment may facilitate oxygen absorption in the Group II films, which will further reduce crystal defects in the II-1 films. The XRD patterns of the films from both groups annealed at 400 °C are similar (Figure 2), which indicates that the lattice order in these films is predominantly determined by the post-deposition annealing. 

**Figure 3:**
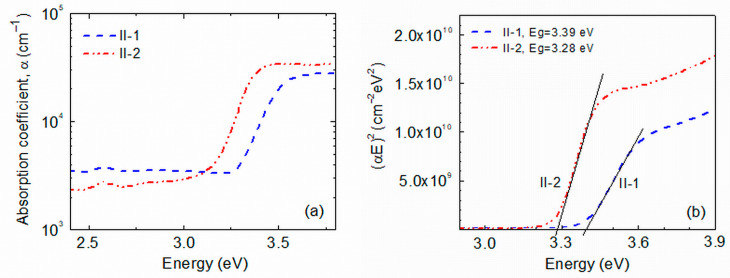
(**a**) Optical absorption coefficients of as-deposited (sample II-1) and annealed (sample II-2) ZnO films from Group II; (**b**) Tauc presentation of the absorption spectra of both ZnO films.

Optical absorption spectroscopy is a frequently used tool for analyzing semiconducting films. The optical absorption spectrum can be used to obtain information about the optical band gap energy (*E*_g_) of semiconductors important for various applications and about the crystal quality and defects or impurities in the material, the degree of material disorder, etc. It is also useful to understand the catalytic behavior of photocatalysts. Optical transmission *T* of Group II ZnO films on Corning 7059 glass substrates was measured. It has been observed that at wavelengths λ > 400 nm, the *T* value of as-prepared samples is around 80% while that of the annealed film is around 90%, being very close to the transmission of the Corning 7059 glass substrate. Figure 3a shows the absorption curves of as-deposited and annealed Group II films. It can be seen that the slope of the Urbach tail in the spectrum of the annealed II-2 film is slightly steeper than that of the as-prepared II-1 films. Moreover, the absorption of the annealed film at energies lower than the optical band gap of ZnO (~3.3 eV) is lower than that of the as-prepared one. These observations speak to the lower density of lattice defects in the annealed sample with energy levels in the forbidden band than in the as-prepared sample. This is in good agreement with the better crystallinity of the annealed films revealed by the X-ray diffraction (XRD) investigations. Additionally, a red-orange photoluminescence band has been observed in the Raman scattering spectra of the films that exhibited a significantly higher intensity in the annealed films than in the as-prepared ones [19]. This result is an indication of a lower density of defects playing the role of centers of non-radiative recombination of photoexcited carriers in the annealed films and also supports the conclusion taken from the optical spectroscopy results. A red-orange photoluminescence band is usually observed in ZnO films fabricated under oxidizing conditions. It has been related to the radiative recombination of photocarriers via interstitial oxygen defects O_i_ [23] or zinc vacancies V_Zn_ [24], which act as acceptors [25]. The hot air treatment of ZnO films, as well as high-temperature annealing in air, are procedures providing oxidizing conditions, and it seems likely that a certain amount of interstitial oxygen and/or zinc vacancies exist in the Group II samples. 

As the grain size in all ZnO films studied here is in the range of 7.5–15.7 nm, no strong carrier confinement was expected in the films [26]. Indeed, no distinguished absorption bands appeared in the optical transmission spectra (Figure 3) and the Tauc method was applied for *E*_g_ estimation. In this method, the dependence of the absorption coefficient, *α*, of the material on the photon energy, *E* = h*ν* (h is the Planck constant, *ν*—photon’s frequency), is described by the relation:

*α* ~ (1/*E*)(*E* − *E*_g_)*^n^.* (1)

Most frequently, *n* = 2 for amorphous materials that have an indirect optical band gap and *n* = 1/2 for crystalline materials with a direct optical band gap. The curve on the Tauc plot (*αE*)^1/n^ vs. *E* should have a linear part, and the optical band gap is determined by extrapolating this linear part to the *x*-axis. Figure 3b shows the Tauc presentation of the films’ absorption in coordinates corresponding to crystalline materials with a direct band gap in which we observed a clear linear section. Values of *E*_g_ = 3.39 eV and 3.28 eV have been obtained for the optical band gap of II-1 and II-2 films, respectively, which are close to the values reported for sol-gel ZnO nanocrystals [27]. Since all films studied are crystalline, the observed difference in the optical band gaps could be due to the observed annealing-induced grain size increase from ~7.5 nm to ~15 nm and/or to the existence of different amounts of organic remains.

### 2.2. Photocatalytic Activity, Recyclability Tests 

**Figure 4:**
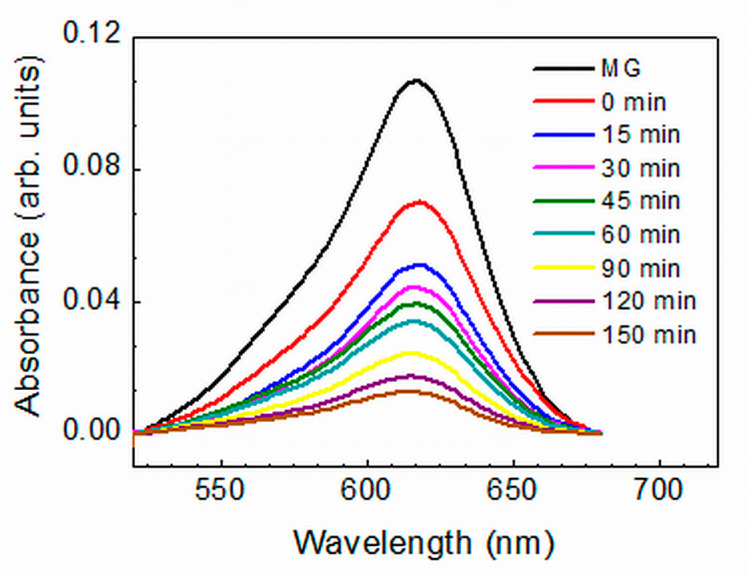
Visible absorbance spectra of Malachite Green in 5 ppm water solution: the MG spectrum was measured in the absence of ZnO film in the solution; the ‘0 min’ spectrum was measured after keeping an annealed II-2 ZnO film in the solution in darkness for 30 min; the other spectra were taken after UV irradiation of this film for different durations denoted in the figure.

The MG photodegradation study in the presence of a tested ZnO film in a water solution was conducted under UV-A irradiation (maximum emission at 365 nm) or VIS light illumination. The photocatalytic efficiency of each layer was determined by measuring the changes in the intensity of the optical absorption maximum at *λ_max_* = 617 nm of Malachite Green dye with irradiation time. Figure 4 shows a set of absorbance spectra obtained for an annealed II-2 sample prepared by applying hot air treatment. For all layers, the intensity of the absorption band decreased with time, indicating a gradual degradation of the dye. The total duration of the tests was determined by the saturation in the photocatalytic activity of the tested films; normally, the duration was 150 min under UV irradiation and 180 min under VIS light illumination. The ratio *f*(*t*) = *C/C*_0_ was used as a measure of the degradation efficiency, where *C*_0_ is the solution absorbance at *λ_max_* = 617 nm at the beginning of light illumination (‘0 min’ spectrum) and *C* is the solution absorbance after irradiation times *t* denoted in the figure. 

#### 2.2.1. Photocatalytic Activity under UV Light Illumination

**Figure 5:**
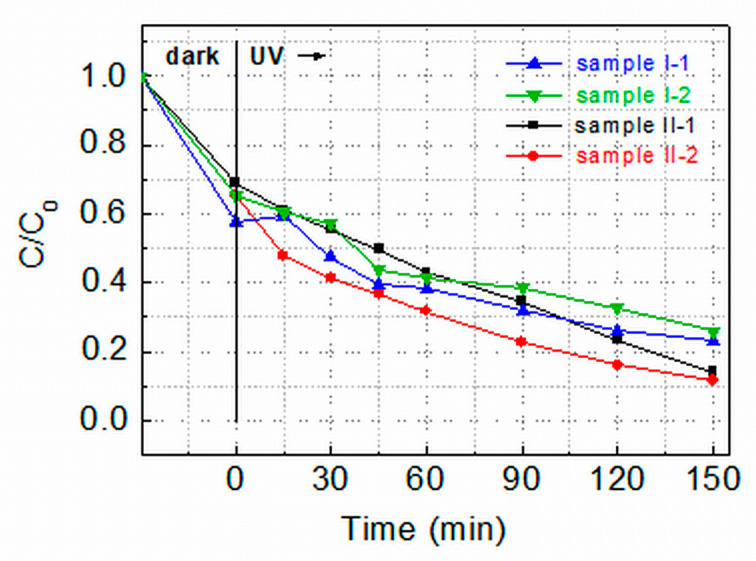
Degradation efficiency of Malachite Green in 5 ppm water solution catalyzed by UV irradiated as-prepared (I-1 and II-1) and annealed (I-2 and II-2) ZnO films deposited by conventional furnace drying in static air at 140 °C (I-1 and I-2) or by applying combined hot air treatment and furnace drying (II-1 and II-2). *C*_0_ is the initial MG concentration in the solution; *C* is the concentration at time *t.* The dashed lines are guides to the eye.

Figure 5 shows a comparison of the degradation efficiency, *C/C*_0_, of as-prepared and annealed films from both groups under UV light irradiation. One can see that after the films’ irradiation for 150 min, the degradation efficiency of the as-prepared film from each group is close to that of the corresponding annealed film. Additionally, the films from Group II demonstrate better efficiency than the films from Group I—the Group II films reduce the MG concentration in the solution down to 11% (II-2)/14% (II-1) of the initial one while the values for the Group I films are 22% (I-1)/25% (I-2). 

**Figure 6:**
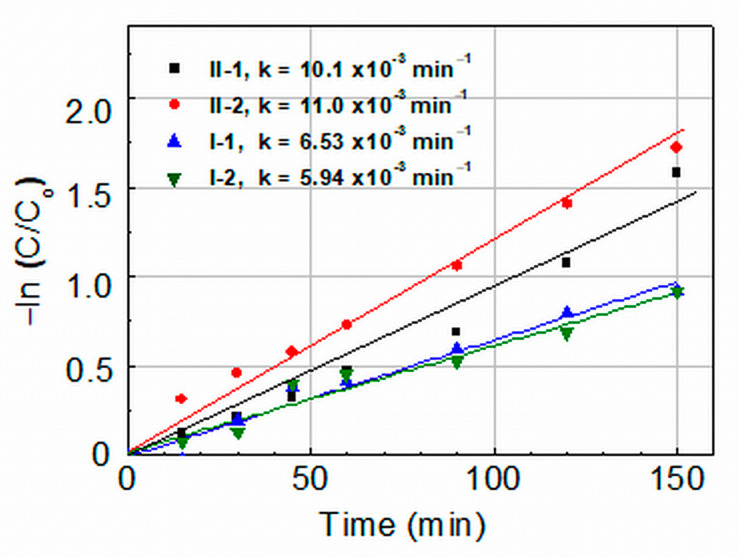
Kinetics and rate constants *k* of MG degradation in 5 ppm water solution catalyzed by UV-irradiated ZnO films prepared by one-step furnace drying in static air at 140 °C (I-1 and I-2) or by combined hot air treatment and furnace drying (II-1 and II-2).

Kinetic curves of the MG degradation are depicted in Figure 6. They show that the photocatalytic degradation of MG under UV light irradiation of all ZnO films follows pseudo first-order kinetics represented by the Langmuir–Hinshelwood equation:

ln(*C/C*_0_) = (−*kt*), (2)

where *k* is the rate constant related to the reactions involving photoexcited electrons (e^−^) and holes (h^+^) on the ZnO film surface; it is a measure of the photocatalytic activity and an important characteristic of catalysts. The rate constants shown in Figure 6 indicate that the Group II films demonstrate significantly higher catalytic activity (*k* ≈ 10–11 × 10^−3^ min^−1^) than the Group I films (*k* ≈ 6–6.5 × 10^−3^ min^−1^). A comparison has been made with rate constants reported in the literature for MG degradation with ZnO photocatalysts, considering that k is affected by various factors (intensity and spectrum of the illuminating light, film thickness/amount of nanocrystalline powder, concentration of dye, pH of the solution, etc.) [14,18]. The inference is that the obtained values of *k* = 10–11 × 10^−3^ min^−1^ are: (i) among the highest reported for the photodegradation of Malachite Green in water solution by pure/unmodified ZnO thin films [28,29] or ZnO nanocrystalline powders [14,30,31], and (ii) comparable with some rate constants determined in photocatalytic MG degradation when using doped or surface-modified ZnO nanostructured films and powders [32,33,34]. It should be noted that normally the total surface of nanocrystalline powders used was significantly larger than the surface of the ZnO thin films studied in our experiments. 

In order to understand the increased photocatalytic activity of Group II films, it should be considered that the surface of these films is smooth and homogeneous, which suggests that the defect density in the near-surface region is lower than in the films of Group I (having a wrinkled surface even after annealing at 400 °C). The XRD data have indicated that the density of lattice defects in the volume of the Group II films is also lower than in the Group I films. Oxygen absorption during the hot air treatment may also reduce the density of oxygen vacancies. An incident UV-A photon with *E > E*g generates a coulombically bound electron–hole pair both in the film volume and on the surface. As it is known [35], in materials with high lattice disorder, geminate or non-geminate recombination may occur before the electron and hole separate into free charge carriers and take part in dye degradation. These recombination processes are due to low mobilities and the small mean-free-path of charge carriers associated with strong carrier scattering. One can suppose that the improved lattice order in Group II layers results in an increased mean-free-path of carriers. This leads to a higher number of photogenerated electrons and holes reaching the film surface and, thus, to better photocatalytic activity. 

The above discussion allows us to connect the conclusion about the existence of fewer crystal defects in the II-2 sample (made from Figure 2a) with the higher photocatalytic activity of this film than the activity of the II-1 film (Figure 5). In this case, the surface of both films is similar (homogeneous and smooth), but the density of the lattice defects in the films’ volume is still different, and this probably led to differences in the recombination and transport of the carriers photogenerated in the volume. Therefore, the number of electrons and holes reaching the II-1 and II-2 film surface could be different. 

#### 2.2.2. Recyclability Tests under UV and VIS Light Irradiation 

**Figure 7:**
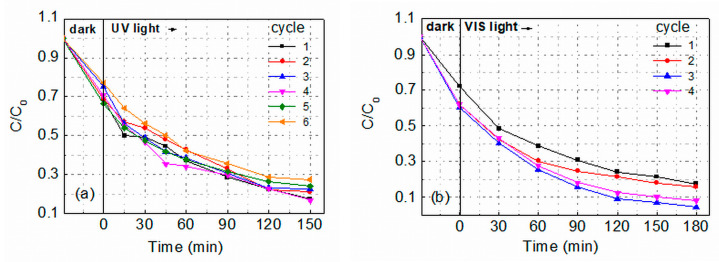
Degradation efficiency (*C/C*_0_) of Malachite Green in 5 ppm water solution catalyzed by UV (**a**) or VIS (**b**) light irradiation of annealed ZnO films prepared by applying hot air treatment (II-2).

The good effect of hot air treatment on the photocatalytic activity of sol-gel ZnO layers raised the question of the influence of this treatment on the reusability of the layers and, therefore, consecutive cycles of reuse were carried out with Group II films under UV and VIS irradiation. After each cycle, the film was removed from the MG dye solution, washed with distilled water, and dried at room temperature in air. Figure 7a,b shows the photocatalytic activity of two II-2 films observed during six and four cycles of MG degradation in 5 ppm water solution upon irradiation with UV or VIS light, respectively.

**Figure 8:**
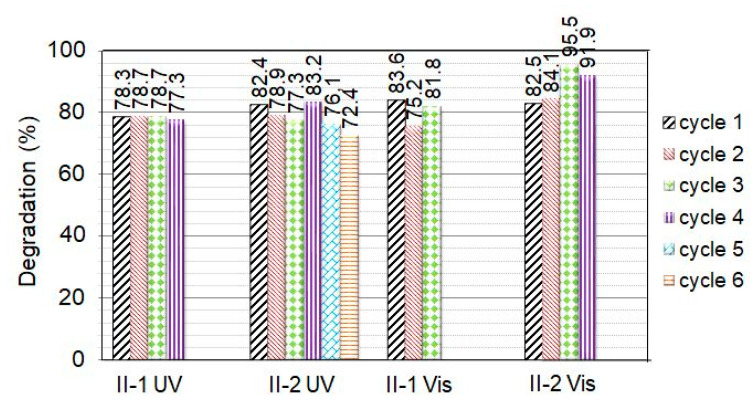
Photocatalytic degradation of Malachite Green after several cycles of UV or VIS light irradiation for 150 min of as-prepared (sample II-1) and annealed (sample II-2) ZnO films prepared by using hot air drying. For each sample, the number of photocatalytic cycles and irradiation type are denoted in the figure.

It is seen that the films ensured successive MG degradation, keeping fairly good activity. Figure 8 summarizes the percentage of degradation (*D*%) obtained for as-prepared (II-1) and annealed (II-2) films; *D* was calculated using the equation:

*D = (C*_0_*− C*_150_*/C*_0_) × 100, (3)

where *C*_150_ is the absorbance at *t* = 150 min. It is seen that under UV irradiation, the photodegradation rate of MG catalyzed by both as-prepared and annealed Group II films shows a slight decrease; i.e., the films retain very good photocatalytic activity during all cycles performed. Interestingly, under VIS light illumination of film II-2, a small D-increase is observed in the second cycle, which becomes more pronounced in the third cycle. Photocurrent measurements performed on II-2 ZnO films at room temperature showed [36] that under VIS light illumination (photon energy *E* < *E*_g_), the photocurrent-to-dark current ratio is quite high (50–400). However, after turning on/off the light, long-time relaxations were observed (lasting several hours). The slow decrease of the current after turning off the light indicates that the recombination of the photoexcited carriers is affected by charge trapping in deep traps. The slow release of absorbed carriers will lead to a higher concentration of free carriers in the next cycle, and the increased concentration could increase the photocatalytic activity until photo-corrosion of the film becomes the dominant process.

It can be seen in Figure 8 that the numbers of successive cycles realized with II-2 films are six and four under UV and VIS light irradiation, respectively. For the as-prepared films, these numbers are four and three. A comparison with literature data [2,16,31,37,38] has shown that although no film doping or other intentional engineering of intrinsic defects was carried out, the reusability of the Group II films is similar to that reported for doped ZnO films, nanocomposites, and nanoparticle assemblies. The number of successive cycles realized when using Group II films speaks to the quite good photo-corrosion resistance of these ZnO films. It is accepted that the photo-dissolution of ZnO in water solution is mainly due to the interaction of photogenerated holes on the ZnO surface with Zn-O bonds and the disassociation of Zn^2+^ from the surface under the reactions:

ZnO + *hv* → ZnO + h^+^ + *e*^−^ (4)

ZnO_s_ + 2*h_s_*^+^ → Zn^2+^_(aq.)_ + 1/2O_2_, (5)

where ZnO represents the ZnO film volume and surface, ZnO_s_ is the ZnO film surface, *hv* is a photon reaching the film, h^+^ and *e*^−^ are a photogenerated hole and electron, *h*_s_^+^ is the hole located on the film surface, Zn^2+^_(aq.)_ is a Zn ion dissolved in the solution. The morphology and surface defects of ZnO, as well as pH and the level of oxygen dissolved in the solution, affect its stability during photocatalysis [5,7,10]. A number of experiments on surface defect engineering have been carried out in order to improve the photo-corrosion resistance of ZnO. A significant enhancement of the photo-corrosion resistance of ZnO nanowire films in water under UV irradiation was obtained after oxygen plasma post-annealing [39], which has been related to the reduction of the oxygen deficiency sites on the film surface and vacancies in the film bulk by the plasma treatment [40]. Correlations between the photo-corrosion of ZnO and lattice relaxation induced by surface vacancies of oxygen and zinc have been reported in [41]. It has been shown that: (i) oxygen vacancies assist photo-corrosion by causing an inward lattice relaxation and the formation of local electric fields around the vacancies, which induce weakness and the cleavage of Zn-O bonds; (ii) adsorption of H_2_, O_2,_ and H_2_O molecules playing the role of electron acceptors leads to photo-corrosion inhibition—they receive excess electrons from oxygen vacancies thus reducing or eliminating the local electric fields. The zinc vacancies also inhibit photo-corrosion by forming an outward lattice relaxation. Above, we have assumed that the hot air treatment facilitates some oxygen absorption during the film preparation and reduces the density of oxygen vacancies in the Group II films. The improved corrosion resistance of these films can be connected with the reduced density of oxygen vacancies on their surface. The post-deposition annealing in air at 400 °C probably had some contribution to the reduction of the density of oxygen vacancies on the film’s surface and the better corrosion resistance of II-2 samples.

**Figure 9:**
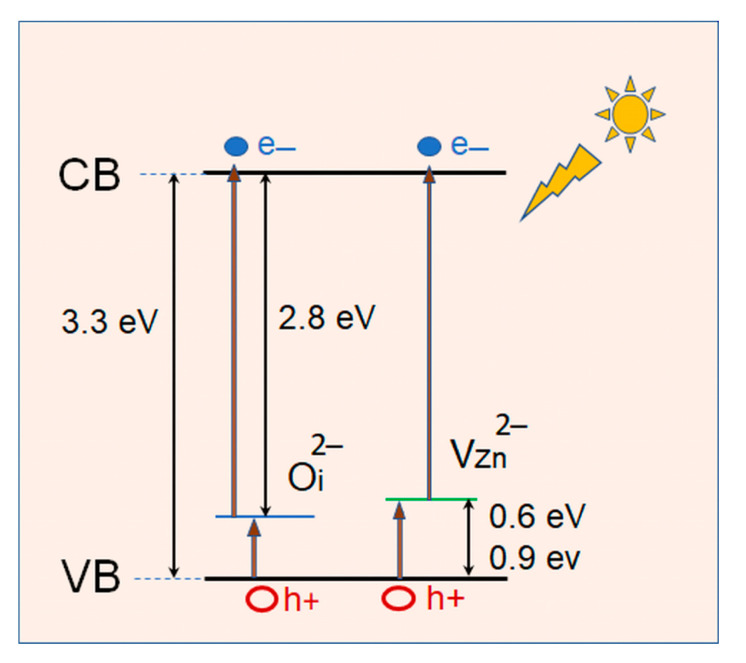
Schematic band diagram of ZnO films with negatively charged O_i_^2−^ [25] and V_Zn_^2−^ [42,43] acceptors.

The results displayed in Figure 8 show that the photocatalytic activity of the Group II films under VIS light illumination is only slightly smaller than that achieved under UV irradiation. It can be supposed that the point defects (V_Zn_ and O_i_) discussed in Section 2.1, acting as acceptors, contribute to the high catalytic activity of these films under visible light illumination. Interstitial oxygen can exist either in electrically inactive “oxygen split” form in semi-insulating and p-type materials or as a deep acceptor at the octahedral interstitial site in n-type materials [25]. When charged with two electrons, the O_i_ acceptor is disposed at an energy depth of around 2.8 eV below the conduction band (CB) bottom, E_c_. For V_Zn,_ a number of theoretical calculations yield a double acceptor level [42]—the (0/−1) transition level is located at around 0.1 eV above the valance band (VB) top E_v_ and the (−1/−2) transition level at around 0.6 eV above E_v_. Values of E_v_ + 0.18 eV and E_v_ + 0.87 eV were also reported in the literature [25,43]. We have found that the dark conductivity of Group II films is much lower than the conductivity of the Group I films [36], which indicates the existence of a considerable density of V_Zn_^2−^/O_i_^2−^ acceptors in Group II films with energy levels situated in the lower half of the band gap. A schematic band diagram of Group II ZnO films based on the cited literature data is shown in Figure 9. We suppose that when the VIS light is turned on, electron transitions occur from the negatively charged acceptors to the conduction band, generating free electrons in the conduction band, which can be further involved in the photocatalytic process. The acceptors from which electrons have been excited may again capture thermally or photoexcited electrons from the valence band, thus generating free holes in the valence band that are also involved in the photocatalytic process. 

## 3. Materials and Methods

### 3.1. Films Preparation

The sol-gel method was used for the preparation of ZnO thin films by using the spin-coating technique. The sol was prepared by dissolving zinc acetate heptahydrate (Zn(CH_3_COO)_2_·7H_2_O, Neochim PLC (Dimitrovgrad, Bulgaria), ≥99.5% purity) in ethanol C_2_H_5_OH (Neochim PLC, 99.98%). Monoethanolamine (MEA, C_2_H_7_NO, ChemPur Feinchemikalien und Forschungsbedarf GmbH (Karlsruhe, Germany), 99.6%) was added as a stabilizing agent; all chemicals are of analytical grade. The molar concentration of each of the two components dissolved in ethanol is 0.5 mol/L. The solution was homogenized with a magnetic stirrer by continuous stirring at 45 °C for 45 min and then aged at room temperature for 24 h. The pH of the resulting solution was 4.8. ZnO films with an approximate size of (1 × 2) cm^2^ were prepared by deposition of a single layer on silicon substrates provided with a thermally grown layer of SiO_2_. The SiO_2_ layer was grown since it allows the performance of electrical measurements by using planar contacts disposed on the top surface of the ZnO layer. Before the film preparation, the substrates were cleaned in an H_2_O_2_ + NH_4_OH solution and then with C_2_H_5_OH (99.98% purity). The full spill time after sol dripping onto the substrates is 30 s at 2800 rpm. To study the optical absorption of the ZnO films, it was necessary to prepare samples on transparent substrates. Layers were successfully made on Corning 7059 glass substrates using hot air drying. However, the attempts to prepare high-quality and stable ZnO layers on these substrates by applying the furnace drying used in the preparation of Group I films on silicon substrates were unsuccessful, and no good optical transmission spectra were obtained. The internal microstrains in the films on Corning 7059 glass substrates are probably high (greater than those in the layers on the silicon substrates revealed from the XRD results) and cause the layer to crack and detach from the substrate.

Two groups of ZnO films were prepared on silicon substrates: Group I was obtained by using convenient furnace drying in static air at 140 °C (413 K) for 15 min (samples I-1); Group II was obtained by applying a two-step drying procedure involving the following steps: first step—treatment with hot air flow (temperature *T*_h_ = 90–95 °C (363–368 K); volume flow rate of 7.5 L/s) directed normally to the sample surface for 5 min; second step—furnace drying in static air at 140 °C for 15 min immediately after the hot air treatment (samples II-1). A number of I-1 and II-1 samples were further annealed in air at 400 °C (673 K) for 60 min (samples I-2 and II-2, respectively). Table 1 summarizes the information about the preparation conditions applied for the production of each type of sample. 

### 3.2. Sample Characterization

The surface morphology of the films was investigated using scanning electron microscopy (SEM) using a JEOL JSM 6390 microscope (Peabody, MA, USA), operated at 20 kV (magnification 2000×, 10,000×, and 20,000×) in regimes of secondary electron image and backscattered electron image. The microscope was equipped with an energy dispersive X-ray spectrometer (EDS), Oxford INCA Energy 350, but the results obtained with the spectrometer were strongly affected by the SiO_2_ film on the top of the Si substrates; the conclusions about the ZnO film composition were not reliable and will not be discussed below. Information on the crystalline structure and lattice order of the films was obtained using X-ray diffraction (XRD) measurements performed on a PANalytical Empyrean diffractometer (Malvern Panalytical Ltd, Malvern, United Kingdom) using the Cu Kα line (λ_Kα_ = 0.15406 nm).

Optical transmission measurements were performed on Group II ZnO films deposited on Corning 7059 glass substrates. An automated UV-VIS-NIR spectrophotometer Lambda 1050 (PerkinElmer, Inc. Waltham, MA, USA) operating under the control of a personal computer (Perkin Elmer software product UV WinLab, version 6.0.2) was used. The measurements were carried out in the range of 270–800 nm in depolarized light. A clear substrate was also measured as a reference. Halogen and deuterium lamps were used as light sources for the VIS and UV regions, respectively. 

An automatic M2000D spectroscopic ellipsometer (Woollam Co., Lincoln, NE, USA) in the transparent wavelength range (600–1000 nm) was used to determine the thickness of the ZnO layers. The experimental ellipsometry Psi and Delta data were fitted using a two-layer model consisting of a silicon substrate covered with a 300 nm thick SiO_2_ and a top layer of ZnO. The Cauchy dispersion equation was applied. It has been obtained that the thickness of the films is in the range of 250–450 nm as the thickness of as-deposited films is considerably larger than that of the corresponding annealed films, i.e., film densification occurs [36].

### 3.3. Photocatalytic Experiments

Photocatalytic experiments were carried out in a water solution of Malachite Green oxalate (MG) azo-dye (Alfa Aesar, Haverhill, MA, USA) with 5 ppm initial concentration and a total volume of 60 mL. All photocatalytic tests were performed at a constant stirring rate of 330 rpm at room temperature (23 ± 2 °C). During each experimental cycle, the ZnO film was placed at 3 mm below the solution’s surface. During the first 30 min of each experimental cycle the film was kept in darkness in order to reach adsorption-desorption equilibrium at the film surface. Afterward, the light was turned on, and the film was illuminated by either UV light from a BLB UV-A 18 W mercury lamp VT (Phillips, Amsterdam, The Netherlands) (light intensity of 0.66 mW/cm^2^) with a maximum emission at 365 nm located at a distance of 10 cm above the vessel with the solution or VIS light from an LB lamp BL 301, 500 W located at a distance of 50 cm from the vessel (light intensity of 16 mW/cm^2^). During the film irradiation, the solution was constantly aerated. The photocatalytic MG degradation was evaluated by taking a part of the solution and measuring the optical absorption of the residual MG concentration using a spectrophotometer UV-1600PC (Thermo-Fisher Scientific, Waltham, MA, USA) (wavelength range 200–800 nm) at time periods of 15 min in the first 60 min of the cycle and then at a period of 30 min until the end of the cycle. After the measurement, the solution was returned to the experimental photocatalytic vessel. 

## 4. Conclusions

Undoped nanocrystalline ZnO thin films were prepared using the sol-gel method with a novel two-step drying procedure involving hot air treatment followed by conventional furnace drying. The effect of the hot air drying on the surface morphology, crystal lattice defects, and photocatalytic behavior in the degradation of Malachite Green azo dye of as-prepared and annealed films was studied by comparing ZnO films prepared by applying conventional furnace drying. The hot-air-treated films demonstrated very good photocatalytic activity with rate constants under UV irradiation of k = 10–11 × 10^−3^ min^−1^, which are considerably higher than those determined for the films prepared using conventional furnace drying (k = 6–6.5 × 10^−3^ min^−1^). The rate constants of the hot-air-treated films are among the best reported in the literature for the photocatalytic degradation of Malachite green using pure/unmodified ZnO thin films and nanocrystalline powder. Based on the results of SEM, XRD, and optical absorption characterization of the layers, it has been assumed that hot air treatment: (i) reduces the density of oxygen vacancies and other lattice defects in the films, thus improving the photocatalytic activity of the films and (ii) facilitates the formation of a certain amount of interstitial oxygen and/or zinc vacancies, which help achieve the high MG degradation efficiency observed under visible light illumination. The good recycling ability of hot-air-treated films (up to six photocatalytic cycles under UV and up to four cycles under VIS light irradiation of annealed films) has been related to the treatment-caused reduction of oxygen vacancies on the surface of the films (the vacancies induce weakness and the cleavage of Zn–O bonds on the film surface and facilitate photo-corrosion). These results indicate that treatment with hot air flow may be used to enhance the multifunctional performance of ZnO films.

## Figures and Tables

**Table 1:** Drying and annealing conditions applied during the sol-gel preparation of the two groups of ZnO thin films.

Film Group	Sample Name	Preparation Steps 1st Step	2nd Step	3rd Step
Group I	I-1	Furnace drying at 140 °C for 15 min		
	I-2	Furnace drying at 140 °C for 15 min	Furnace annealing at 400 °C for 60 min	
Group II	II-1	Hot air drying at ~95 °C for 5 min	Furnace drying at 140 °C for 15 min	
	II-2	Hot air drying at ~95 °C for 5 min	Furnace drying at 140 °C for 15 min	Furnace annealing at 400 °C for 60 min

## Data Availability

All data are included in the article.
